# 1,8-Cineole Affects Agonists-Induced Platelet Activation, Thrombus Formation and Haemostasis

**DOI:** 10.3390/cells10102616

**Published:** 2021-10-01

**Authors:** Kahdr A. Alatawi, Divyashree Ravishankar, Pabitra H. Patra, Alexander P. Bye, Alexander R. Stainer, Ketan Patel, Darius Widera, Sakthivel Vaiyapuri

**Affiliations:** 1 School of Pharmacy, University of Reading, Reading RG6 6UB, UK; K.M.A.Alatawi@pgr.reading.ac.uk (K.A.A.); divyasri.april86@gmail.com (D.R.); drphpatra@gmail.com (P.H.P.); d.widera@reading.ac.uk (D.W.); 2 School of Biological Sciences, University of Reading, Reading RG6 6UB, UK; a.bye@reading.ac.uk (A.P.B.); Alexander.Stainer@porterhousemedical.com (A.R.S.); ketan.patel@reading.ac.uk (K.P.)

**Keywords:** 1,8-cineole, platelets, collagen, haemostasis, thrombosis, platelet reactivity, signalling

## Abstract

1,8-cineole, a monoterpenoid is a major component of eucalyptus oil and has been proven to possess numerous beneficial effects in humans. Notably, 1,8-cineole is the primary active ingredient of a clinically approved drug, Soledum^®^ which is being mainly used for the maintenance of sinus and respiratory health. Due to its clinically valuable properties, 1,8-cineole has gained significant scientific interest over the recent years specifically to investigate its anti-inflammatory and antioxidant effects. However, the impact of 1,8-cineole on the modulation of platelet activation, thrombosis and haemostasis was not fully established. Therefore, in this study, we demonstrate the effects of 1,8-cineole on agonists-induced platelet activation, thrombus formation under arterial flow conditions and haemostasis in mice. 1,8-cineole largely inhibits platelet activation stimulated by glycoprotein VI (GPVI) agonists such as collagen and cross-linked collagen-related peptide (CRP-XL), while it displays minimal inhibitory effects on thrombin or ADP-induced platelet aggregation. It inhibited inside-out signalling to integrin αIIbβ3 and outside-in signalling triggered by the same integrin as well as granule secretion and intracellular calcium mobilisation in platelets. 1,8-cineole affected thrombus formation on collagen-coated surface under arterial flow conditions and displayed a minimal effect on haemostasis of mice at a lower concentration of 6.25 µM. Notably, 1,8-cineole was found to be non-toxic to platelets up to 50 µM concentration. The investigation on the molecular mechanisms through which 1,8-cineole inhibits platelet function suggests that this compound affects signalling mediated by various molecules such as AKT, Syk, LAT, and cAMP in platelets. Based on these results, we conclude that 1,8-cineole may act as a potential therapeutic agent to control unwarranted platelet reactivity under various pathophysiological settings.

## 1. Introduction

Cardiovascular diseases (CVD) specifically thrombotic conditions such as ischemic stroke and heart attacks are the leading cause of death around the modern world. Platelets are small anucleated circulating blood cells and they play an essential role in the maintenance of haemostasis through blood clotting upon vascular injury [1]. However, the inappropriate activation of platelets during pathological conditions such as upon the rupture of atherosclerotic plaque results in the formation blood clots (thrombi) within the vasculature leading to reduced/blocked blood supply to vital organs such as the heart and brain. Therefore, platelets act as a powerful therapeutic target to prevent/treat thrombotic diseases and indeed, anti-platelet drugs such as aspirin and clopidogrel play predominant roles in the treatment, management, and prevention of these conditions. However, the currently available anti-platelet drugs present serious side effects such as gastrointestinal toxicity, bleeding, and tolerance in some patients. Hence, there is an unmet clinical need for more effective and safer anti-platelet drugs to prevent and treat thrombotic diseases [2].

Naturally available small molecules derived from plants have remained as a major source of active pharmaceutical ingredients for drug discoveries [3]. While the impact of a broad spectrum of flavonoids on the modulation of platelet activation has been established in detail, the research on the modulatory effects of terpenoids on platelets is limited. Essential oils are volatile plant secondary metabolites containing a complex mixture of monoterpenoids, sesquiterpenoids and other oxygenated hydrocarbons [4,5]. They play critical roles in many industries such as to produce cosmetics, perfumes, food materials and pharmaceuticals [6,7]. Essential oils from various plants possess numerous chemicals with therapeutic effects such as anti-inflammatory properties [8]. 1,8-cineole (also known as ‘eucalyptol’), a principal constituent of eucalyptus oil has been increasingly used in medical applications due to its wider pharmacological effects [9,10]. Chemically, it is a bicyclic monoterpene [11] and has been reported to possess anti-inflammatory, antimicrobial and antioxidant effects in several pathological conditions [12]. Indeed, 1,8-cineole is the active ingredient of Soledum^®^, a clinically approved medication for the treatment of respiratory tract diseases [12,13]. Some in vitro studies have shown that 1,8-cineole exhibits anti-inflammatory activity by inhibiting the production of certain cytokines in monocytes when stimulated with lipopolysaccharides (LPS) [14]. 1,8-cineole is also able to reduce hypersecretion of mucus in airways during asthma [14]. Furthermore, 1,8-cineole inhibits the activities of specific pro-inflammatory mediators of NF-κB-signalling by preventing the nuclear translocation of p65 [15]. Intrigued by the broad range of beneficial effects of 1,8-cineole, here, we demonstrate its effects in the modulation of platelet activation, thrombus formation and haemostasis.

## 2. Results

### 2.1. 1,8-. Cineole Largely Inhibits Platelet Aggregation Induced by GPVI Agonists

**Figure 1:**
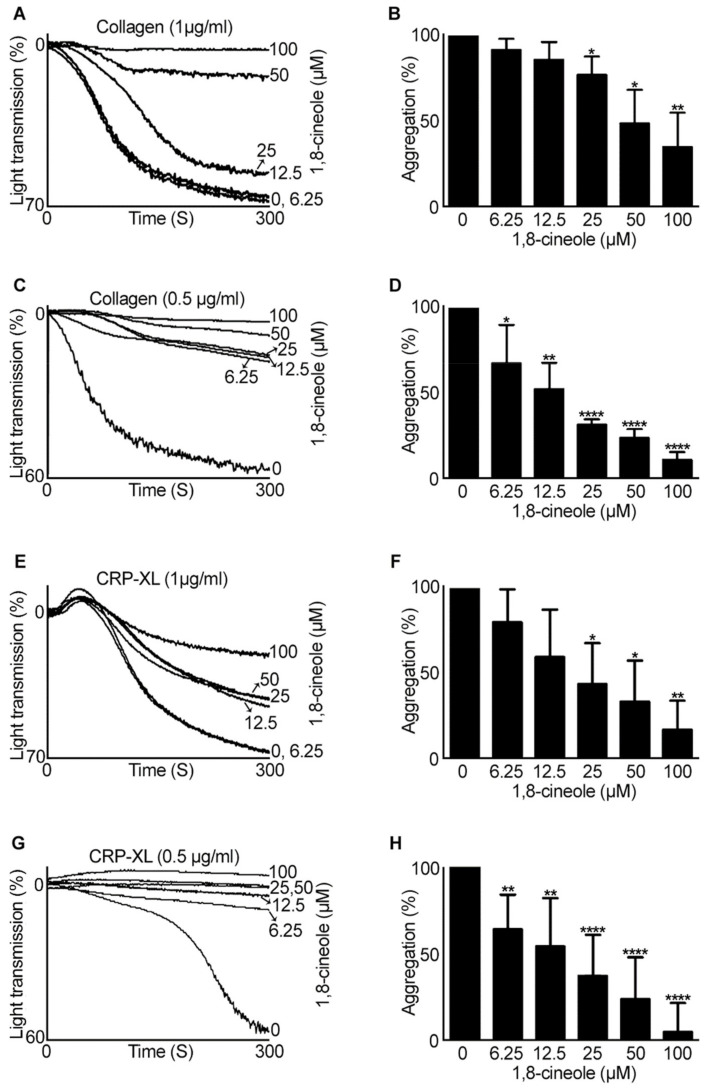
Effect of 1,8-cineole on GPVI agonists-stimulated aggregation in human isolated platelets. A vehicle control [0.1% (*v/v*) ethanol] or various concentrations of 1,8-cineole were incubated with human isolated platelets for 5 min prior to stimulation of aggregation with 1 µg/mL (**A**,**B**) or 0.5 µg/mL (**C**,**D**) collagen, and 1 µg/mL (**E**,**F**) or 0.5 µg/mL (**G**,**H**) CRP-XL. The level of aggregation was monitored for 5 min in an optical aggregometer. The aggregation traces shown are representative of five separate experiments. The percentage of aggregation for 1,8-cineole-treated samples was calculated by considering the level of aggregation obtained with the vehicle control (0) as 100%. Data represent mean ± SEM (*n* = 5). The *p* values shown (* *p* < 0.05, ** *p* < 0.01, *** *p* < 0.001 and **** *p* < 0.0001) are as calculated by one-way ANOVA followed by Bonferroni post hoc test.

To determine the impact of 1,8-cineole on the modulation of platelet activation, aggregation assays were performed using various agonists. Human isolated platelets (4 × 10^8^ cells/mL) were incubated with a vehicle control [0.01% (*v/v*) ethanol] or various concentrations of 1,8-cineole (6.25 µM–100 µM) for five minutes prior to stimulation with different concentrations of GPVI agonists such as collagen and cross-linked collagen-related peptide (CPR-XL). At higher concentrations (50 and 100 µM), 1,8-cineole significantly inhibited (by around 50–70%) 1 µg/mL collagen-activated platelet aggregation (Figure 1A,B). However, when the concentration of collagen was reduced to 0.5 µg/mL, 1,8-cineole displayed significant inhibitory effects at all the concentrations used (Figure 1C,D). Notably, 100 µM of 1,8-cineole has completely inhibited collagen-induced platelet aggregation, while the other concentrations have shown around 30-80% inhibition. Since collagen binds to both GPVI and integrin α2β1 receptors to activate platelets, a selective agonist for GPVI, CRP-XL was then used to determine the precise effects of 1,8-cineole via this receptor. Similar to collagen, CRP-XL (1 µg/mL) induced platelet aggregation was largely reduced by higher concentrations of 1,8-cineole (Figure 1E,F), although at a lower (0.5 µg/mL) concentration of CRP-XL, all the concentrations of 1,8-cineole inhibited platelet aggregation in a concentration-dependent manner (Figure 1G,H). 

**Figure 2:**
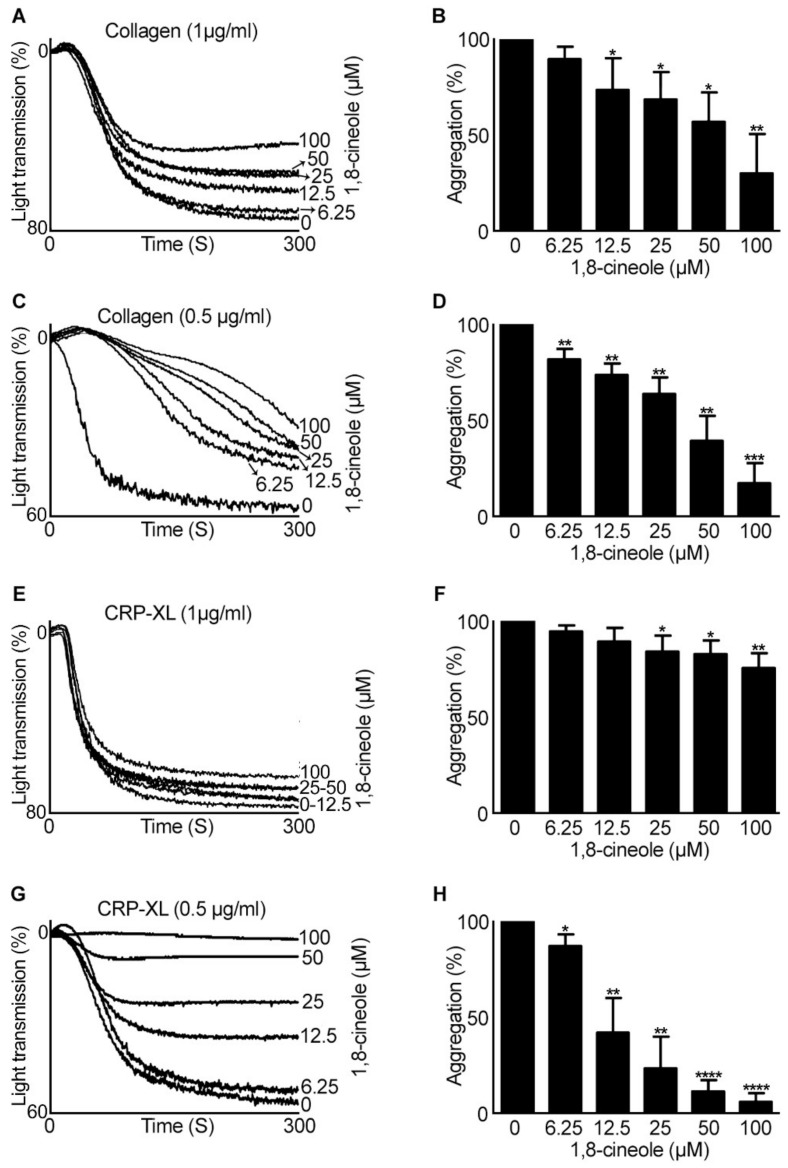
Impact of 1,8-cineole on GPVI agonists-stimulated aggregation in human PRP. Human PRP was treated with a vehicle control [0.1% (*v/v*) ethanol] or various concentrations of 1,8-cineole for 5 min prior to stimulation of aggregation with 1 µg/mL (**A**,**B**) or 0.5 µg/mL (**C**,**D**) of collagen, and 1 µg/mL (**E**,**F**) or 0.5 µg/mL (**G**,**H**) of CRP-XL. The level of aggregation was monitored for 5 min by optical aggregometry. The aggregation traces shown are representative of five separate experiments. The percentage of aggregation for 1,8-cineole-treated samples was calculated by considering the level of aggregation obtained with the vehicle control (0) as 100%. Data represent mean ± SEM (*n* = 5). The *p* values shown (* *p* < 0.05, ** *p* < 0.01, *** *p* < 0.001 and **** *p* < 0.0001) are as calculated by one-way ANOVA followed by Bonferroni post hoc test.

Following the determination of the inhibitory effects of 1,8-cineole on isolated platelets, its effects in the modulation of platelet activation was determined in the presence of plasma proteins using human platelet-rich plasma (PRP). Similar to its effects in isolated platelets, 1,8-cineole did not show large effects on 1 µg/mL collagen (Figure 2A,B) and CRP-XL (Figure 2E,F)-induced platelet aggregation. However, when the concentration of collagen (Figure 2C,D) and CRP-XL (Figure 2G,H) was reduced to 0.5 µg/mL, 1,8-cineole inhibited platelet aggregation at all the concentrations tested. 

**Figure 3:**
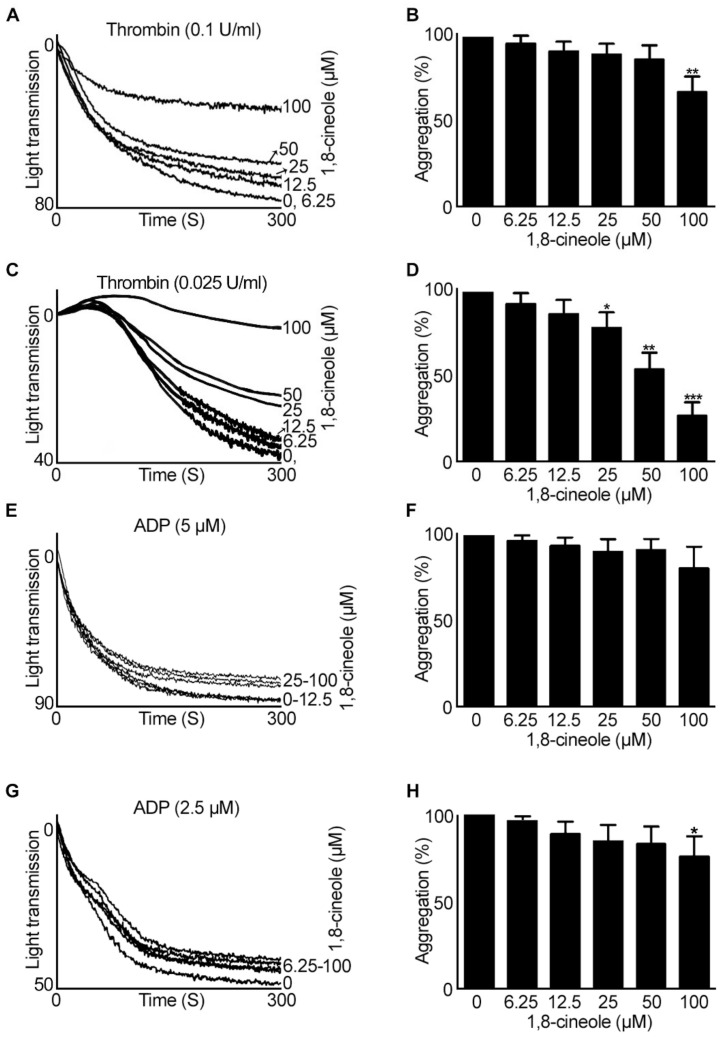
Effect of 1,8-cineole on thrombin- and ADP-stimulated aggregation in human platelets. Human isolated platelets were incubated with a vehicle control [0.1% (*v/v*) ethanol] or various concentrations of 1,8-cineole for 5 min prior to stimulation of aggregation with 0.1 U/mL (**A**,**B**) or 0.025 U/mL (**C**,**D**) thrombin. Similarly, human PRP was incubated with various concentrations of 1,8-cineole for 5 min prior to inducing platelet aggregation with 5 µM (**E**,**F**) or 2.5 µM (**G**,**H**) ADP. The level of aggregation was monitored for 5 min by optical aggregometry. The aggregation traces shown are representative of four separate experiments. The percentage of aggregation for 1,8-cineole-treated samples was calculated by considering the level of aggregation obtained with the vehicle control (0) as 100%. Data represent mean ± SEM (*n* = 4). The *p* values shown (* *p* < 0.05, ** *p* < 0.01 and *** *p* < 0.001) are as calculated by one-way ANOVA followed by Bonferroni post hoc test.

Furthermore, to analyse if 1,8-cineole is able to inhibit platelet aggregation induced by agonists that stimulate platelet activation via G-protein-coupled receptors (GPCRs), aggregation assays using thrombin in human isolated platelets (4 × 10^8^ cells/mL) and ADP in human PRP were performed. Except at a concentration of 100 µM, 1,8-cineole did not affect thrombin (0.1 U/mL) (Figure 3A,B) induced platelet aggregation. However, when the concentration of thrombin was reduced to 0.025 U/mL, 1,8-cineole inhibited platelet aggregation at 25 µM and above (Figure 3C,D). ADP (Figure 3E–H)-induced platelet aggregation was not affected by 1,8-cineole at any of the concentrations used except a small (around 20–30%) inhibition observed at 100 µM when lower concentration (2.5 µM) of ADP was used. Together, these results suggest that 1,8-cineole mainly inhibits platelet aggregation induced by collagen and CRP-XL although at higher concentrations it affects other agonists-induced platelet activation. 

### 2.2. Inside-out Signalling to Integrin αIIbβ3 Is Affected by 1,8-Cineole

**Figure 4:**
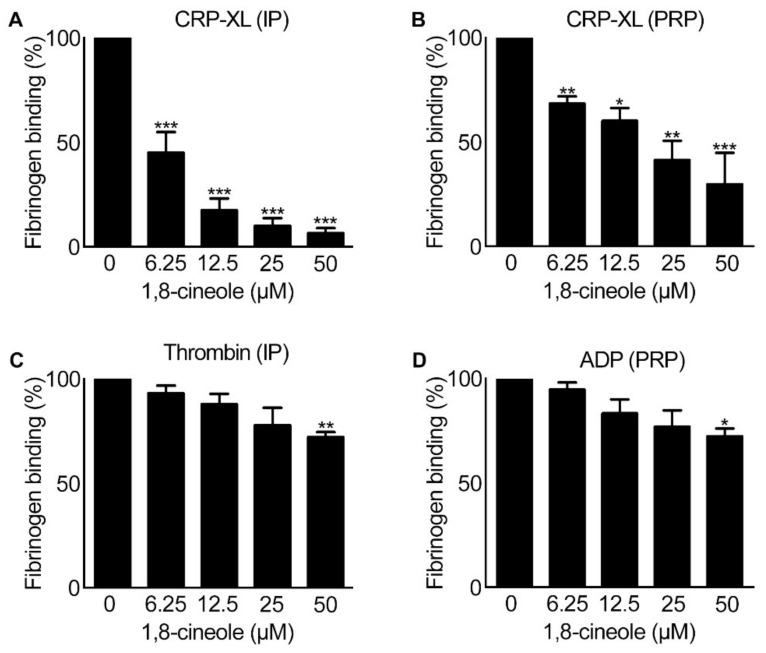
Effect of 1,8-cineole on inside-out signalling to integrin αIIbβ3 in human platelets. Human isolated platelets (IP) or PRP were incubated with a vehicle control [0.01% (*v/v*) ethanol] or various concentrations of 1,8-cineole for 5 min prior to addition of CRP-XL (0.5 μg/mL) (**A**,**B**), thrombin (0.025 U/mL) (**C**) or ADP (2.5 μM) (**D**) and further incubation for 20 min at room temperature. The level of fibrinogen binding (as a marker for inside-out signalling to integrin αIIbβ3) on the platelet surface was quantified using FITC-conjugated anti-human fibrinogen antibodies by flow cytometry. The bar graph indicates the percentage of fibrinogen binding as calculated with respect to the vehicle (0) control (considered as 100%). Data represent mean ± SEM. (*n* = 5). The *p* values shown (* *p* < 0.05, ** *p* < 0.01 and *** *p* < 0.001) are as calculated by one-way ANOVA followed by Bonferroni post hoc test.

Platelet aggregation is dependent on the conformational changes to integrin αIIbβ3 (a highly abundant platelet surface receptor [16]) induced by inside-out signalling in platelets in order to transform the receptor from a low-affinity state to a high affinity binding state for fibrinogen and von Willebrand factor (vWF) [16,17]. Therefore, to determine the effect of 1,8-cineole on this critical event, the level of fibrinogen binding as a marker for inside-out signalling to integrin αIIbβ3 was measured in platelets upon stimulation with CRP-XL (0.5 µg/mL), thrombin (0.025 U/mL) and ADP (2.5 μM). 1,8-cineole showed significant reduction in the level of fibrinogen binding in both human isolated platelets (4×10^8^ cells/mL) (Figure 4A) and PRP (Figure 4B) upon stimulation with CRP-XL at all the concentrations used. However, when thrombin (in isolated platelets) and ADP (in PRP) were used as agonists, 1,8-cineole inhibited fibrinogen binding only at a higher concentration (50 μM). These results demonstrate that 1,8-cineole is able to affect the inside-out signalling to integrin αIIbβ3, and thereby controls the level of fibrinogen binding on the platelet surface and subsequent platelet aggregation. 

### 2.3. 1,8-. Cineole Affects Granule Secretion in Platelets

**Figure 5:**
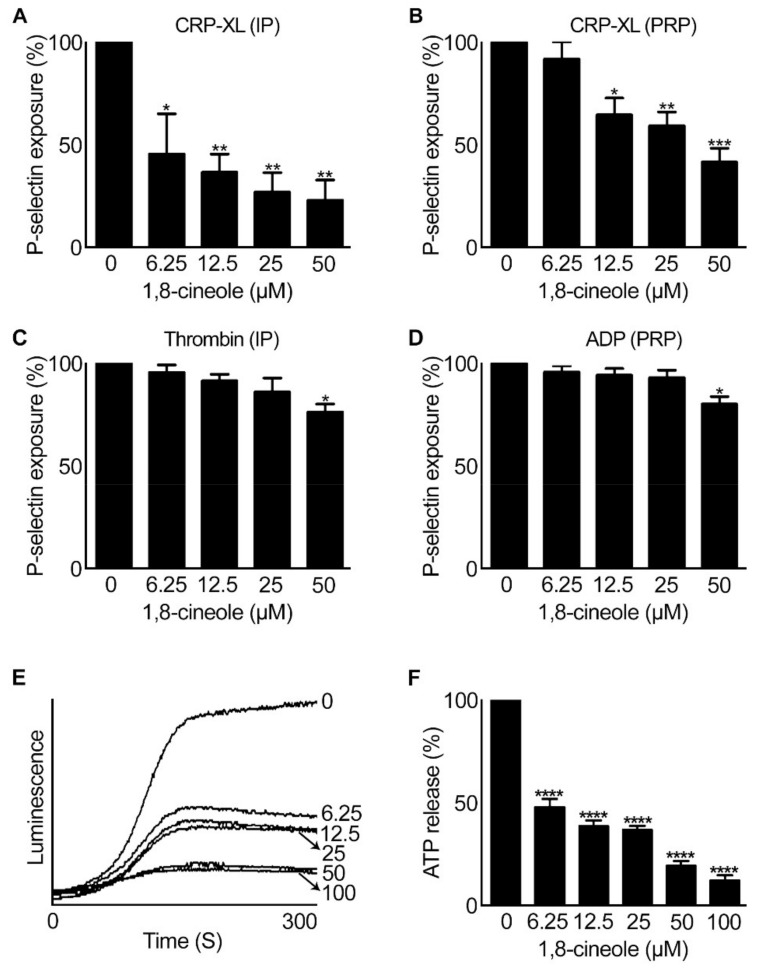
Impact of 1,8-cineole on granule secretion in human platelets. Human isolated platelets (IP) or PRP were incubated with a vehicle control [0.01% (*v/v*) ethanol] or various concentrations of 1,8-cineole for 5 min prior to the addition of CRP-XL (0.5 μg/mL) (**A**,**B**), thrombin (0.025 U/mL) (**C**) or ADP (2.5 μM) (**D**) and further incubation for 20 min at room temperature. The level of α-granule secretion in platelets was determined by quantifying the amount of P-selectin exposed (as a marker for α-granule secretion) on the platelet surface upon activation using PE/Cy5-conjugated anti-human P-selectin antibodies by flow cytometry. The bar graphs show the effects of various concentrations of 1,8-cineole on α-granule secretion in human isolated platelets or PRP upon stimulation with different agonists. Moreover, the effect of 1,8-cineole on dense granule secretion was quantified by measuring the level of ATP release upon activation of platelets. The human isolated platelets were incubated with different concentrations of 1,8-cineole or a vehicle control [0.01% (*v/v*) ethanol] for 5 min in the presence of the Chrono-lume luciferin-luciferase reagent and the level of ATP released upon platelet activation with 0.5 µg/mL CRP-XL was monitored using lumi-aggregometry. The traces (**E**) shown are representative of four separate experiments. The cumulative data (**F**) shown demonstrate the effect of 1,8-cineole on dense granule secretion in platelets as calculated by considering the level of ATP release observed with the vehicle control as 100%. Data represent mean ± SEM. (*n* = 4). The *p* values shown (* *p* < 0.05, ** *p* < 0.01, *** *p* < 0.001 and **** *p* < 0.0001) are as calculated by one-way ANOVA followed by Bonferroni post hoc test.

Platelets release several effector molecules from their granules upon stimulation with agonists to augment platelet activation and thrombus formation [18]. Platelets contain different granules such as α-granules, dense granules, and lysosomes. α-granules stores mainly proteins such as fibrinogen, vWF and P-selectin, whereas small organic molecules such as ADP, ATP and serotonin are stored in dense granules [19,20]. Hence, the effect of 1,8-cineole on α-granule secretion in platelets was assessed by measuring the level of P-selectin exposure on the platelet surface upon stimulation with CRP-XL (0.5 µg/mL), thrombin (0.025 U/mL) and ADP (2.5 μM) using flow cytometry. The level of P-selectin exposure was significantly reduced in the presence of 1,8-cineole at all concentrations (6.25 µM–50 µM) tested in human isolated platelets (4×10^8^ cells/mL) (Figure 5A) and at concentrations between 12.5 µM–50 µM in human PRP (Figure 5B) when CRP-XL was used. While thrombin (Figure 5C) or ADP (Figure 5D) was used, 1,8-cineole inhibited P-selectin exposure only at a higher concentration of 50 µM. Similarly, the effect of 1,8-cineole on dense granule secretion upon platelet activation was analysed by measuring the level of ATP secretion using lumi-aggregometer. Human isolated platelets (4×10^8^ cells/mL) were incubated with luciferase-luciferin chrono-lume reagent in the presence and absence of different concentrations of 1,8-cineole prior to stimulation with CRP-XL (0.5 µg/mL) to measure the level of ATP released. Indeed, 1,8-cineole has displayed significant inhibitory effects on dense granule secretion at all the concentrations tested (Figure 5E,F). These results demonstrate the effects of 1,8-cineole on the modulation of granule secretion in platelets. 

### 2.4. 1,8-. Cineole Inhibits Intracellular Calcium Mobilisation in Platelets

**Figure 6:**
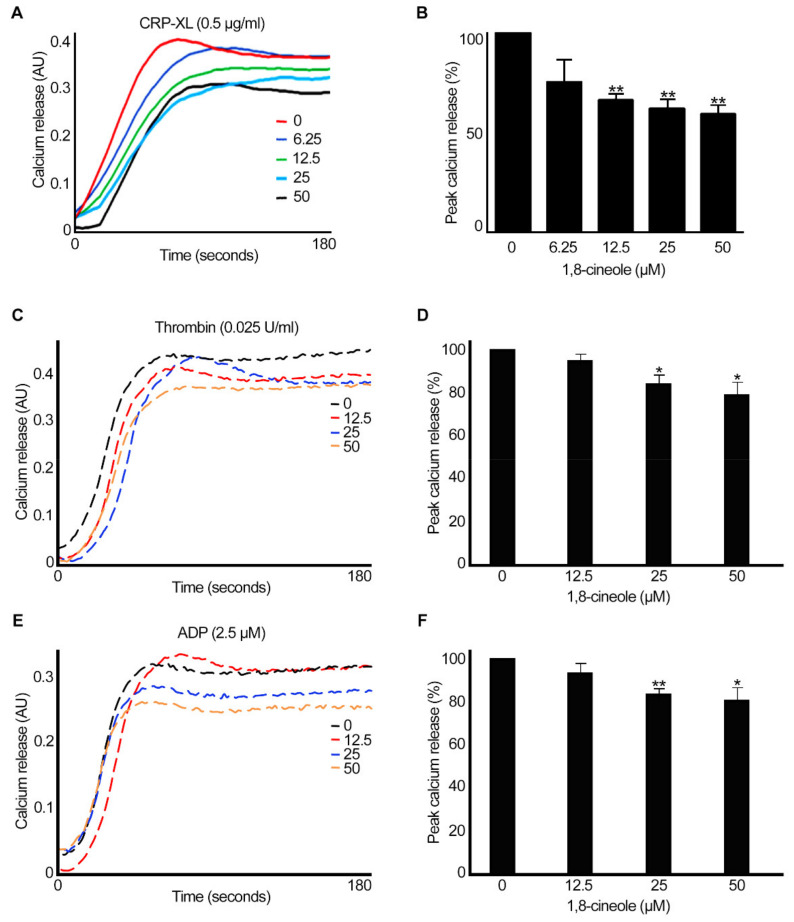
Effect of 1,8-cineole on intracellular calcium mobilisation in human platelets. Human PRP (**A**,**E**) or isolated platelets (**C**) treated with Fluo-4 AM dye were incubated with a vehicle control or various concentrations of 1,8-cineole for 5 min prior to stimulation of calcium release with CRP-XL (0.5 µg/mL) (**A**,**B**), thrombin (0.025 U/mL) (**C**,**D**) or ADP (2.5 µM) (**E**,**F**). The level of calcium release was monitored for 3 min by spectrofluorimetry. The traces shown are representative of four separate experiments. The cumulative data were calculated by taking the peak calcium released in the vehicle control as 100%. Data represent mean ± SEM. (*n* = 4). The *p* values shown (* *p* < 0.05 and ** *p* < 0.01) are as calculated by one-way ANOVA followed by Bonferroni post hoc test.

Calcium is a critical mediator of platelet activation, and its levels are largely increased in platelet cytoplasm via release from intracellular stores (dense tubular system) and influx from plasma [21]. Therefore, the impact of 1,8-cineole on the mobilisation of intracellular calcium levels was analysed using Fluo 4-calcium sensitive dye in human PRP or isolated platelets (for thrombin) (4 × 10^8^ cells/mL) upon activation with CRP-XL (0.5 µg/mL), thrombin (0.025 U/mL) or ADP (2.5 µM) by spectrofluorimetry. The pre-incubation of platelets with different concentrations of 1,8-cineole has affected the peak calcium level in platelets upon stimulation with CRP-XL (Figure 6A, 6B). When thrombin (0.025 U/mL) (Figure 6C,D) or ADP (2.5 µM) (Figure 6E,F), the level of calcium was only affected to a smaller extent (around 20%) by higher concentrations of 1,8-cineole. These results demonstrate that 1,8-cineole can affect the intracellular calcium mobilisation which is a critical event during platelet activation and subsequent thrombus formation.

### 2.5. Integrin αIIbβ3-Mediated Outside-in Signalling Is Affected by 1,8-cineole

**Figure 7:**
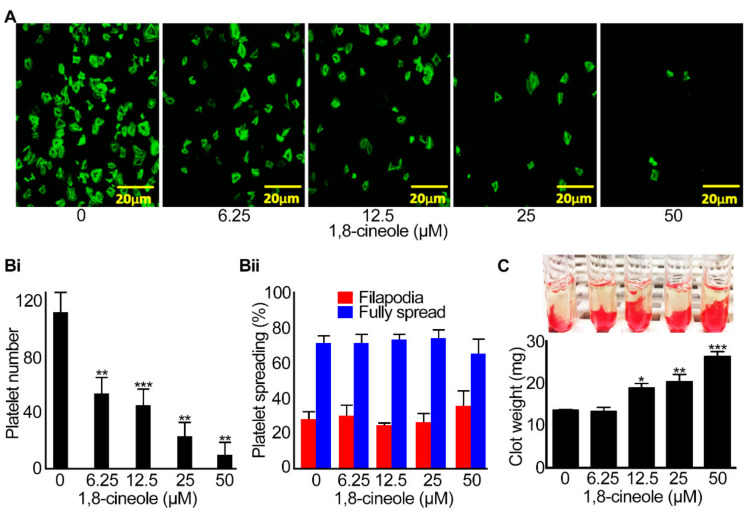
Effect of 1,8-cineole on integrin αIIbβ3-mediated outside-in signalling in human platelets. Human isolated platelets (at a density of 2x10^7^ cells/mL) were incubated with a vehicle control (0) or different concentrations of 1,8-cineole for 5 min and added onto fibrinogen- (100 μg/mL) coated coverslips and allowed them to spread for 45 min. Following fixation with 0.2% (*v/v*) formyl saline followed by permeabilisation with 0.2% (*v/v*) Triton X-100, the platelets were stained with Alexa Fluor 488-conjugated phalloidin for visualisation. Platelet spreading was analysed using a 100x oil immersion lens on a Nikon A1-R confocal microscope. Ten random images of view were recorded and for each sample, random locations on the slides were analysed. The number of platelets at different stages of spreading was determined by analysing the images using ImageJ. (**A**) representative images captured at 45 min of platelet spreading in the absence and presence of different concentrations of 1,8-cineole. (**Bi**) the cumulative data showing the number of platelets adhered to fibrinogen in control and 1,8-cineole treated samples. (**Bii**), the relative percentage of adhered platelets that progressed to filopodia and full spread stages on fibrinogen at 45 min. Data represent mean ± SEM (*n* = 4 individual experiments using platelets obtained from four volunteers, and for each, 10 images were used for analysis). (**C**) to determine the impact of 1,8-cineole on clot retraction, human PRP was treated with various concentrations of 1,8-cineole prior to addition of 1 U/mL thrombin and monitoring of clot retraction for 2 h. The images shown are representative of four separate experiments. The data shown were calculated by measuring the remaining clot weights after 2 h of retraction. Data represent mean ± SEM (*n* = 4). The *p* values shown (* *p* < 0.05, ** *p* < 0.001 and *** *p* < 0.001) are as calculated by one-way ANOVA followed by Bonferroni post hoc test.

Integrin αIIbβ3-mediated outside-in signalling plays critical roles to induce platelet spreading and at a later stage, clot retraction to facilitate wound healing [22,23]. To determine the effect of 1,8-cineole on the outside-in signalling mediated by integrin αIIbβ3, platelet spreading on fibrinogen-coated glass surface and the clot retraction assay were performed. Human isolated platelets (2 × 10^7^ cells/mL) were incubated with different concentrations (6.25 µM–50 µM) of 1,8-cineole prior to adding them to human fibrinogen-coated glass cover slips and allowing them to spread for 45 min. The analysis of confocal microscopy images demonstrates that 1,8-cineole significantly affects the number of platelets adhered on fibrinogen-coated surfaces (Figure 7A,Bi). At the concentration of 50 µM of 1,8-cineole, only a small number of platelets were able to adhere to fibrinogen. However, the progression of adhered platelets to filopodia formation and complete spreading was not affected by 1,8-cineole (Figure 7Bii), which may be due to significantly less adhered platelets in 1,8-cineole treated samples compared to the controls. To determine the impact of 1,8-cineole on clot retraction, human PRP was incubated various concentrations of 1,8-cineole (6.25–50 μM) prior to initiating clot formation by the addition of 1 U/mL thrombin. The rate of clot retraction was monitored over 2 h by taking images at every 30 min. The effect of 1,8-cineole on clot retraction was analysed by measuring the remaining clot weight after 2 h. As expected, the clot size was completely retracted in the vehicle control, whereas the clot retraction was reduced in 1,8-cineole-treated samples with significant reduction observed at 12.5 μM and above (Figure 7C). Together, these data suggest that 1,8-cineole is able to influence integrin αIIbβ3-mediated outside-in signalling in platelets.

### 2.6. 1,8-Cineole Reduces Thrombus Formation under Arterial Flow Conditions

**Figure 8:**
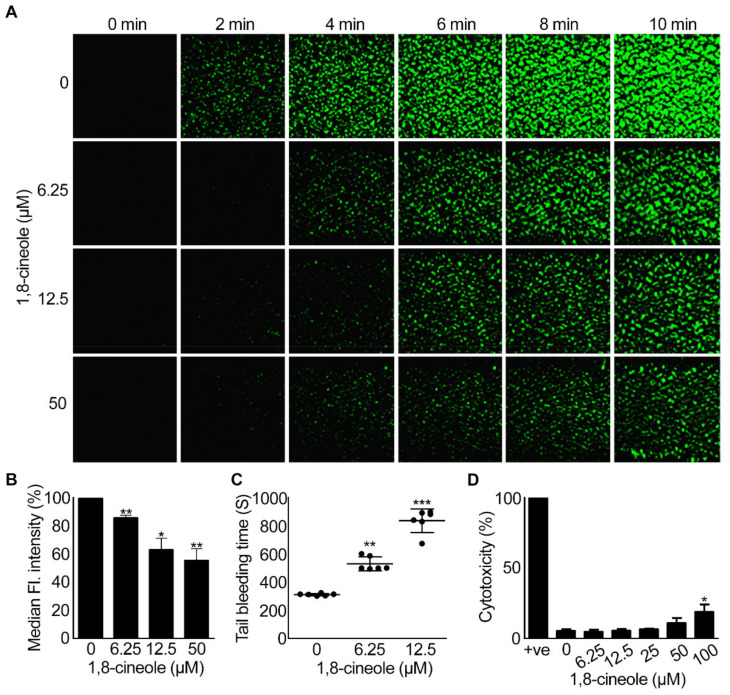
Impact of 1,8-cineole on thrombus formation and haemostasis. DiOC6 (a lipophilic dye) (5 μM)-labelled human whole blood was incubated with a vehicle or different concentrations of 1,8-cineole for 5 min and perfused through microfluidic channels (Vena8 BioChips) coated with collagen (400 μg/mL). Thrombus formation was observed using a 20 × objective on a Nikon A1-R confocal microscope, with images captured every 30 s up to 10 min (**A**). Quantified data represent median fluorescence intensity of thrombi formed at 10 min in control and 1,8-cineole-treated samples as calculated using NIS elements software (Nikon) and normalised to the level of median fluorescence intensity obtained for thrombi at 10 min in the vehicle treated sample (**B**). Data represents mean ± SEM (*n* = 3). The p values (* *p* < 0.05, and ** *p* < 0.01) shown are as calculated by one-way ANOVA with Dunnett’s post hoc test. (**C**) Effect of 1,8-cineole on haemostasis in mice was analysed using a tail bleeding assay. Mice (*n* = 6 per group) were anaesthetised and a vehicle control [0.01% (*v/v*) ethanol] or 1,8-cineole (6.25 µM or 12.5 µM) was administered via femoral artery. After 5 minutes of incubation, 3 mm of tail tip was dissected, and the tail tip was placed in sterile PBS. The time for cessation of bleeding was measured up to 20 minutes. Data represent mean ± SEM (*n* = 6). The *p* values shown (** *p* < 0.01 and *** *p* < 0.001) are as calculated by non-parametric Kruskal–Wallis test. (**D**) To determine whether 1,8-cineole exerts any cytotoxic effects on human platelets, human isolated platelets were exposed to a positive control, a vehicle control [0.1% (*v/v*) ethanol] or various concentrations of 1,8-cineole for 30 min and the amount of LDH released (a marker for cytotoxicity) was measured at 490 nm and 650 nm using spectrophotometry. The maximum LDH release obtained with the positive control was taken as 100% and the level of LDH release for 1,8-cineole treated samples was calculated accordingly. Data represent mean ± SEM (*n* = 3). The *p* value shown (* *p* < 0.05) was calculated by one-way ANOVA with post hoc Dunnett’s test.

Platelet aggregation following vascular injury culminates in thrombus formation in order to seal the damaged area and prevent bleeding [1]. To determine the impact of 1,8-cineole on whole blood (i.e., in the presence of other blood cells and plasma proteins), thrombus formation on collagen-coated Vena8 biochips was analysed under arterial flow conditions. DiOC6-labelled human whole blood was incubated with various concentrations of 1,8-cineole prior to infusion over collagen-coated capillaries in Vena8 biochips and the level of thrombus formation was monitored for 10 min by taking images at every 30 s. 1,8-cineole at concentrations of 6.25 μM, 12.5 μM and 50 μM significantly inhibited the platelet adhesion, thrombus growth, volume (Figure 8A) and the fluorescence intensity (Figure 8B) of thrombi formed. These data suggest that 1,8-cineole is able to affect platelet activation and subsequent thrombus formation in whole blood as similar to their inhibitory effects in isolated platelets and PRP.

### 2.7. 1,8-. Cineole Affects Haemostasis in Mice 

Haemostasis is a normal physiological response of the body to prevent excessive bleeding upon vascular injury [24]. To investigate the effect of 1,8-cineole on haemostasis, a tail-bleeding assay was performed in mice in the presence of a vehicle control or various concentrations (6.25 μM and 12.5 μM) of 1,8-cineole. Following the clipping of 3 mm tail tip, the bleeding time was monitored. The vehicle-treated mice bled for around 300 s, whereas the administration of 1,8-cineole extended the bleeding time to around 500 s at 6.25 μM and 800 s with 12.5 μM (Figure 8C). These results indicate that 1,8-cineole affects the haemostasis in mice even at a low concentration of 6.25 μM to a modest level.

### 2.8. 1,8-. Cineole Is Not Cytotoxic to Platelets at Lower Concentrations 

Finally, to determine whether 1,8-cineole pharmacologically inhibits platelet activation, or it exerts any cytotoxic effects, lactate dehydrogenase (LDH) cytotoxicity assay was performed. Human isolated platelets (4 × 10^8^ cells/mL) were incubated with different concentrations of 1,8-cineole (6.25 μM–100 μM) along with a positive control, and the amount of LDH released was measured as a marker for cytotoxicity using a spectrophotometer. 1,8-cineole was found to be non-toxic up to 50 μM concentration, however, a low level of cytotoxicity was observed at 100 μM concentration (Figure 8D). This result indicates that the inhibitory effects of 1,8-cineole up to 50 μM are due to its pharmacological effects in platelets rather than its cytotoxicity. However, caution should be taken when 1,8-cineole is used at or above 100 μM as it is likely to cause cytotoxicity at these concentrations.

### 2.9. 1,8-. Cineole Affects Various Signalling Pathways in Platelets

**Figure 9:**
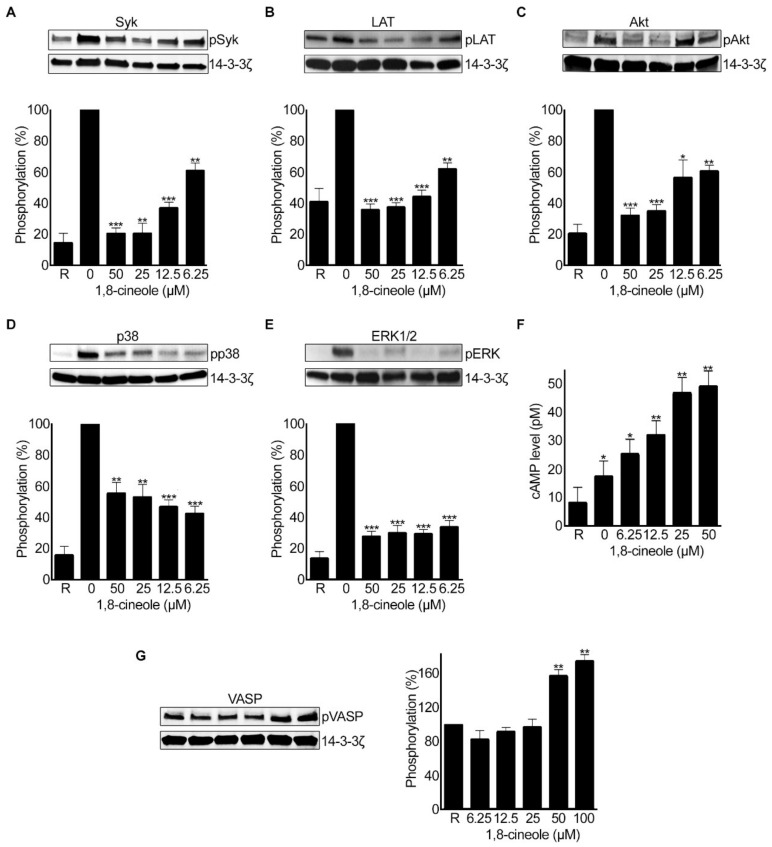
Effect of 1,8-cineole on specific signalling proteins and cAMP levels in platelets. Human isolated platelets (4 × 10^8^ cells/mL) were treated with a vehicle control (0) or various concentrations of 1,8-cineole for 5 min before stimulation with CRP-XL (0.5 µg/mL) for 5 min in an aggregometer at 37°C. Then, the cells were lysed using reducing sample treatment buffer and analysed in SDS-PAGE followed by immunoblots using various phospho-specific antibodies. The impact of 1,8-cineole on the phosphorylation of pSyk (Y525/526) (**A**), pLAT (Y200) (**B**), pAKT (S473) (**C**), pp38 (**D**), and pERK1/2 (**E**) was analysed using selective phospho-specific antibodies for these proteins in immunoblots. (**F**) the level of cAMP in platelets that were treated with a vehicle control or various concentrations of 1,8-cineole was measured using a cAMP ELISA kit in line with the manufacturer’s instructions. Data represent mean ± SEM. (*n* = 4). (**G**), the phosphorylation of VASP (S157) was analysed using platelets that were treated with a vehicle control or different concentrations of 1,8-cineole. The level of 14-3-3ζ was detected as a loading control in all these blots. The blots shown are representative of three separate experiments. Data represent mean ± SEM (*n* = 3), normalised to loading control. The *p* values shown (* *p* < 0.05, ** *p* < 0.01 and *** *p* < 0.001) are as calculated by one way-ANOVA followed by Bonferroni’s correction for multiple comparisons.

1,8-cineole has been reported to modulate various signalling pathways (e.g., cytokine production and NF-κB activity) that are involved in inflammatory responses [14,15]. Here, as 1,8-cineole largely inhibited GPVI-mediated platelet activation, the effect of 1,8-cineole on the phosphorylation of key downstream proteins in GPVI signalling pathway was investigated using human isolated platelets (4 × 10^8^ cells/mL) by immunoblot analysis. 1,8-cineole affected the phosphorylation of Syk (Figure 9A) and LAT (Figure 9B), which are key regulators of GPVI signalling pathway. Then, the impact of 1,8-cineole on the phosphorylation of AKT, which is a critical downstream effector molecule of phosphoinositide 3 kinase (PI3K) signalling was evaluated. Indeed, 1,8-cineole inhibited the phosphorylation of AKT at all the concentrations tested (Figure 9C). To determine the impact of 1,8-cineole on mitogen-activated protein kinase (MAPK) signalling pathways, the phosphorylation of p38 and ERK1/2 was analysed using immunoblots. Similar to other signalling proteins, 1,8-cineole affected the phosphorylation of both p38 (Figure 9D) and ERK1/2 (Figure 9E) at all the concentrations tested. To further explore the other targets for 1,8-cineole in platelets, the level of cAMP was measured in the absence and presence of various concentrations of this molecule without an agonist. 1,8-cineole has increased the level of cAMP (Figure 9F) and the phosphorylation of VASP which is a substrate for cAMP-dependent protein kinase (PKA) (Figure 9G). Together, these data demonstrate that 1,8-cineole is able to affect not only GPVI signalling pathway, but also it influences MAPK and cAMP-mediated signalling in platelets. However, we cannot rule out the possibility of its impact on other signalling molecules/pathways in platelets as it may target multiple pathways in platelets.

## 3. Discussion

Over the last few decades, extensive research has been performed on medicinal plants to identify and develop new drugs with reduced side effects for various human diseases [3]. Since platelets act as a powerful therapeutic target to control thrombotic diseases [2], numerous plant-derived small molecules have been tested to determine their ability to inhibit platelet activation and thrombosis without any adverse effects on haemostasis. Indeed, flavonoids such as quercetin [25,26], catechin [27,28], tangeretin [29] and nobiletin [30,31] were extensively studied for their inhibitory effects in platelets. However, research on investigating the anti-platelet effects of essential oils that contain terpenoids is highly limited. Notably, essential oils and their chemical constituents have shown to exhibit various pharmacological effects [5]. For example, eugenol, a major component of clove oil has been reported to inhibit the oxidation of low-density lipoproteins thereby it reduces the development of atherosclerosis [32]. α-curcumene, a major constituent of turmeric essential oil exerts triglyceride-lowering activity on serum as well as liver triglycerides [33]. Interestingly, the essential oil from lavender has been reported to inhibit platelet aggregation induced by agonists such as collagen, ADP, arachidonic acid and U46619 [34]. 1,8-cineole is a major active component of eucalyptus oil and thymus herb-derived essential oils [12]. 1,8-cineole has previously been shown to possess numerous beneficial effects including antioxidant and anti-inflammatory properties [12,13]. However, the effects of 1,8-cineole on the modulation of platelet function have remained largely unexplored. Hence, in this study, the ability of 1,8-cineole to inhibit platelet activation and thrombus formation was investigated. 

Similar to several flavonoids [29,30] and eugenol [35], 1,8-cineole inhibits platelet activation induced by agonists such as collagen and CRP-XL. A concentration-dependent inhibition of 1,8-cineole was observed in aggregation assays that were performed with human isolated platelets upon stimulation with CRP-XL and collagen. These effects were largely present when human PRP was used although a small reduction in their activities was observed. The binding of small molecules to plasma proteins was previously reported for various plant-derived compounds [29,36]. For example, tangeretin a flavonoid rich in lemon peel [29] and quercetin which is abundant in red onions [37] were shown to bind plasma proteins to an extent. Therefore, the binding of 1,8-cineole to plasma proteins may reduce its bioavailability. While the level of inhibition observed with the low concentrations of 1,8-cineole was prominent when collagen and CRP-XL were used as agonists, it only inhibited thrombin or ADP-induced platelet aggregation at higher concentrations. When the concentration of thrombin was reduced, the effect of 1,8-cineole was more prominent at 25 µM and above. Similar to our findings with 1,8-cineole, eugenol (from nutmeg oil) was found to inhibit collagen-induced platelet aggregation with reduced effects on ADP- and thrombin-induced aggregation [35,38]. Another study has demonstrated that the essential oil of cloves in which eugenol was a major component, inhibits collagen-induced platelet aggregation but was not able to cause a significant inhibition on ADP-induced platelet aggregation [39]. The essential oil of lavender has been reported to inhibit platelet aggregation induced by collagen, thromboxane receptor agonist (U46619), arachidonic acid and ADP on PRP [34]. In the same study, the antiplatelet effect of the main components of lavender essential oil [linalyl acetate (36.2%), linalool (33.4%), camphor (7.6%) and 1,8-cineole (5.8%)] were also investigated [34]. The inhibitory effects of 1,8-cineole observed on platelet aggregation in our study are also similar to the effects observed with several naturally occurring flavonoids including tangeretin [29] and nobiletin [30]. These observations strongly indicate that 1,8-cineole may mainly affect GPVI-induced platelet activation pathway, although its effects on other pathways may be minimal. 

The stimulation of platelets by agonists induce inside-out signalling that transforms the conformation of the extracellular domain of integrin αIIbβ3 resulting in an increase in its binding affinity for plasma fibrinogen to facilitate aggregation [16]. As similar to aggregation, 1,8-cineole inhibits the level of fibrinogen binding on platelet surface induced by CRP-XL and other agonists. This indicates its ability to affect inside-out signalling to integrin αIIbβ3 which results in reduced platelet aggregation. Moreover, 1,8-cineole inhibited both α- and dense granule secretion in platelets upon stimulation with agonists. As components released from dense granules (e.g., ADP) and α-granules (e.g., vWF and fibrinogen) are essential regulators of secondary activation of platelets and thrombus formation [20], this inhibition by 1,8-cineole suggests its ability to suppress the positive feedback cascades that lead to a rapid and large activation of platelets during thrombus formation. Similar to 1,8-cineole, other essential oils such as elemicin and eugenol isolated from *Cymbopogon ambiguus* are volatile monoterpenoids with potent anti-inflammatory effects [40]. Both elemicin and eugenol have been reported to possess anti-platelet effects by inhibiting ADP-induced secretion of serotonin in human platelets. Eugenol exhibited potent inhibitory activity on ADP-induced platelet aggregation compared to aspirin [40]. Another study has demonstrated the inhibitory effects of eugenol on human PRP aggregation induced by arachidonic acid, ADP and collagen with prominent inhibitory effects on arachidonic acid-induced platelet aggregation [41]. They also suggested that eugenol has an inhibitory effect on cyclooxygenase (COX) activity by inhibiting thromboxane A_2_ production similar to aspirin. In addition, 1,8-cineole has affected intracellular calcium mobilisation in platelets. The elevation of calcium levels through release from intracellular stores and entry from outside via influx mechanisms is critical during platelet activation [21]. However, 1,8-cineole has shown to affect the calcium levels following agonists-induced platelet activation suggesting that it may affect platelet reactivity at multiple stages. 

Integrin αIIbβ3-mediated outside-in signalling amplifies a range of cellular events that are essential for platelet functions such as spreading and clot retraction [23]. 1,8-cineole significantly inhibited the adhesion of platelets (with no effects of filapodia formation and complete spreading) on fibrinogen-coated surface and clot retraction. Platelet spreading is critical to allow platelet adhesion at the injury site and to provide surface for clotting cascades to take place, which finally results in generation of thrombin, another powerful activator of platelets [42]. The effect of 1,8-cineole on platelet spreading is similar to several other flavonoids including tangeretin [29], nobiletin [30] and chrysin [43]. The clot retraction is another assay where the significance of integrin αIIbβ3-mediated outside-in signalling can be assessed [44]. The retraction process of the fibrin mesh is primarily driven by integrin αIIbβ3 which facilitates the interaction between fibrinogen bound on the surface of platelets and myosin-actin cytoskeleton inside the platelets [23]. 1,8-cineole inhibited clot retraction with increasing clot weights when concentrations were increased. Likewise, essential oil of lavender inhibited the clot retraction induced by thrombin in PRP [34]. Similar to 1,8-cineole, other essential oils such as oils of *Ocotea quixos* [45] and *Foeniculum vulgare* [46] reduced the clot retraction rate indicating their significance in integrin αIIbβ3-mediated outside-in signalling.

The inhibition of numerous functions associated with platelet activation by 1,8-cineole suggests its ability to subsequently modulate thrombus formation. Therefore, the impact of 1,8-cineole on whole human blood was investigated by in vitro thrombus formation assay under arterial flow conditions. Indeed, 1,8-cineole reduced thrombus formation significantly by inhibiting platelet adhesion, thrombi number and volume over time. In contrast to other assays where isolated platelets or PRP were used, here the whole blood was used. Hence, this demonstrates the ability of 1,8-cineole to inhibit platelet function in the presence of plasma proteins and other blood cells. The prolonged exposure of this compound to platelets in the circulation may cause modest inhibition over time to prevent the unwarranted activation of platelets. Finally, the impact of 1,8-cineole on the modulation of haemostasis in mice was determined by tail bleeding assay. Here, 1,8-cineole (at 12.5 μM and 6.25 μM) has shown to moderately extend the bleeding time in mice, which reflects the interaction between platelets and damaged blood vessel, leading to the formation of a haemostatic plug. Furthermore, the effect of 1,8-cineole on bleeding time could also be due to its vasodilation effects as reported in a previous study [47]. However, the impact of 1,8-cineole on the modulation of haemostasis in humans under diverse pathophysiological scenarios should be investigated. Interestingly, 1,8-cineole was found to be non-cytotoxic to platelets up to 50 µM, and only a concentration of 100 µM has caused a mild (significant) toxic effect although this is a supraphysiological concentration which may not be achieved therapeutically. 

The molecular mechanistic studies indicated that 1,8-cineole may have multiple targets in platelets as similar to several other plant-derived small molecules. 1,8-cineole inhibits the phosphorylation of Syk and LAT which are involved in GPVI signalling pathway [48]. This may reflect on the inhibitory effects of 1,8-cineole selectively on collagen and CRP-XL-induced platelet activation. During clot retraction process, the initial binding of cytoskeletal myosin depends on the phosphorylation of integrin β3 subunit with important downstream roles of Src and PLCγ2 [49]. The inhibitory effects of 1,8-cineole on clot retraction can be through the decreased activity of PLCγ2 and Src-family kinase. Furthermore, it inhibits the phosphorylation of AKT at Serine 473, which is a downstream effector molecule of PI3K that plays a key role in platelet activation [50]. A previous study has shown that 1,8-cineole inactivates AKT in mice that exhibit hepatic lesions analogous to non-alcoholic steatohepatitis where the levels of AKT are upregulated [51]. Studies have indicated that 1,8-cineole inactivates AKT and survivin in human colon cancer cell lines [52] and it inhibits GSK-3 in nasal polyps in patients with chronic rhinosinusitis [53]. Previous studies that were aimed to investigate the anti-inflammatory activity of 1,8-cineole have also indicated that 1,8-cineole interferes with various signalling pathways [14,15]. For example, in human umbilical vein endothelial cells (HUVECs), it has reported that 1,8-cineole mainly mediates its inhibitory effects via the NF-κB signalling pathway resulting in the suppression of pro-inflammatory cytokine release induced by bacterial LPS. Moreover, 1,8-cineole has shown to inhibit MAPKs specifically p38 and ERK1/2 which are also critically important to regulate various cellular events in platelets [54]. Interestingly, 1,8-cineole increased the level of cAMP in platelets in the absence of an agonist. The increased level of cAMP was also observed in platelets when treated with different flavonoids [29]. The increase in cAMP levels by 1,8-cineole is likely due to its direct effects on adenylate cyclase (which regulates cAMP production) or phosphodiesterase (PDE), which controls the levels of cAMP in platelets to maintain them under resting conditions in circulation [2]. Together, these data demonstrate that 1,8-cineole inhibits platelet activation via various signalling pathways. Although this study has investigated various aspects of platelet activation using 1,8-cineole, the complete understanding of its mechanisms of action was not established in this single study. Therefore, further studies are required to establish the complete landscape of the mechanisms through which 1,8-cineole modulate platelet activation and its impact under pathological circumstances (using animal models and/or blood samples obtained from human patients with CVD). Moreover, the impact of 1,8-cineole in the modulation of thrombosis and haemostasis under in vivo conditions in mice and humans should be investigated in future to demonstrate its clinical applications.

## 4. Materials and Methods 

### 4.1. Materials Used

The clinical grade (99%) 1,8-cineole (C80601) and all other regular chemicals were purchased from Sigma Aldrich, UK. Horm collagen (Nycomed, Linz, Austria), CRP-XL (obtained from Prof. Richard Farndale at the University of Cambridge), thrombin (T6884) and ADP (01905) (Sigma Aldrich, Gillingham, UK) were used as platelet agonists in this study. Fluo-4 NW calcium assay (F36206) and LDH cytotoxicity assay kits (88953) were purchased from ThermoFisher Scientific, Gloucester, UK. Vena8 biochips were obtained from Cellix Limited, Dublin, Ireland. 12 weeks old mice purchased from Envigo, London, UK were used for tail bleeding assay. The phosphospecific antibodies [pVASP S157 (3111S), pAKT S473 (4058S), pP38 T180/Y182 (4511S) and pSyk Y525/526 (2711S)] and anti-14-3-3ζ (sc-293415) used in this study were from Cell Signalling technology and Santacruz Biotechnology, respectively. Similarly, pLAT Y200 (ab68139) and pERK1/2 (ab201015) were obtained from Abcam, Cambridge, UK. The Cy5 labeled anti-rabbit (A10523) and Cy3 labeled anti-mouse (A10521) antibodies were obtained from ThermoFisher Scientific, Gloucester, UK. 

### 4.2. Platelet Preparation

The preparation of human platelets was performed using standard protocols as described previously [43,55]. All the experiments were performed in accordance with relevant institutional and national guidelines. A written informed consent was obtained from human volunteers according to the procedures approved by the University of Reading Research Ethics Committee (UREC 17/17). The blood was drawn from healthy, aspirin-free human volunteers via venipuncture into vacutainers containing 3.2% (*w/v*) sodium citrate as an anticoagulant. 

Platelet-rich plasma (PRP) preparation: Blood samples were centrifuged at 102 g for 20 min at 20°C. The resultant supernatant (PRP) was collected and rested for 30 min at 30°C prior to using them in aggregation, flow cytometry and clot retraction assays. 

Preparation of isolated platelets: 50 mL of whole blood was mixed with 7.5 mL of ACD [acid citrate dextrose; 20 g/L glucose, 25 g/L sodium citrate and 15 g/L citric acid] and centrifuged at 102 g for 20 min at 20 °C. The supernatant (PRP) was aspirated and to this 3 mL of ACD and 10 µL of prostaglandin I_2_ (PGI_2_) (125 µg/mL) were added prior to centrifuging at 1413 g for 10 min at 20 °C. The resulting platelet pellet was washed by resuspending in modified Tyrodes-HEPES buffer (25 mL) [2.9 mM KCl, 134 mM NaCl, 0.34 mM Na_2_HPO_4_.12H_2_O, 1 mM MgCl_2_, 12 mM NaHCO_3_, 20 mM HEPES, pH 7.3] in the presence of 10 µL of PGI_2_ (125 µg/mL) and centrifuging at 1413 g for 10 min. The resulting platelet pellet was finally resuspended in modified Tyrodes-HEPES buffer at a density of 4 × 10^8^ cells/mL and rested for 30 min before use in aggregation, flow cytometry and immunoblot assays. 

### 4.3. Preparation of 1,8-Cineole 

Clinical grade 1,8-cineole was diluted in modified Tyrodes-HEPES buffer with 5% ethanol to the desirable concentrations for assays and the final concentration of ethanol in platelets was maintained at 0.01% (*v/v*). A vehicle control [ethanol at a concentration of 0.01% (*v/v*)] was included in all the experiments and this concentration did not affect the platelet function. 

### 4.4. Aggregation and ATP Release Assays 

Platelet aggregation assays were performed using optical aggregometer (Chrono-Log, Havertown, PA, USA) as described by us previously [55,56]. Platelets (445 µL) were incubated with different concentrations of 1,8-cineole or a vehicle control [0.01% (*v/v*) ethanol] (5 µL) for 5 min at 37°C. The samples were then activated with 50 µL of different concentrations of ADP, collagen, CRP-XL or thrombin and the level of platelet aggregation was monitored for 5 min.

ATP release was determined as a measure for dense granule secretion in platelets using the luciferin-luciferase reagent by lumi-aggregometry (Chrono-Log, Havertown, PA, USA). Briefly, platelets (395 µL) were incubated with Chrono-Lume reagent (50 µL) for 2 min at 37°C. After this, 5 µL of various concentrations of 1,8-cineole was added and incubated was for 5 min prior to activation with 50 µL of agonist as stated above.

### 4.5. Flow Cytometry-Based Assays

The human isolated platelets or PRP were incubated with different concentrations of 1,8-cineole or a vehicle control for 5 min in the presence of FITC-labelled anti-human fibrinogen antibodies (Dako, Thetford, UK) and PECy5-labelled CD62P (P-selectin) antibodies (BD Biosciences, Berkshire, UK). Platelets were then activated with CRP-XL (0.5 µg/mL), ADP (2.5 µM using PRP) or thrombin (0.025 U/mL using isolated platelets) for 20 min at room temperature. Following this, 0.2% (*v/v*) formyl saline was added to fix the platelets and the levels of fibrinogen binding (a marker for inside-out signalling to integrin αIIbβ3) and P-selectin exposure (a marker for α-granule secretion) were measured by flow cytometry (Accuri C6, BD Biosciences, Berkshire, UK). The median fluorescence intensity was used to assess the levels of fibrinogen binding and P-selectin exposure on the platelet surface. The level of fluorescence obtained with the vehicle control was taken as 100% to calculate the levels of fibrinogen binding and P-selectin exposure in 1,8-cineole treated samples.

### 4.6. Calcium Mobilisation 

The intracellular calcium levels in platelets were measured using Fluo-4 AM calcium-sensitive dye (Life Technologies, UK), which binds free intracellular calcium. 2 mL of human PRP (or isolated platelets for thrombin) were loaded with 2 mL (2 µM final concentration) of Fluo-4 AM and incubated for 45 min at 30 °C in the dark. The isolated platelets or PRP loaded with Fluo-4 AM were incubated with a vehicle control [(0.01% (*v/v*) ethanol] or different concentrations (6.25, 12.5, 25, and 50 μM) of 1,8-cineole before activating with 0.5 μg/mL CRP-XL, ADP (2.5 µM) or thrombin (0.025 U/mL). The level of fluorescence intensity was measured by a Fluostar Optima plate reader (BMG Labtech, Ortenberg, Germany) at 37 °C for 5 min using an excitation wavelength of 480 nm, and emission at 520 nm. The data were analysed by measuring the percentage of the maximum level of calcium was released in all the samples.

### 4.7. Clot Retraction Assay

Human PRP (200 μL) and red blood cells (5 μL) were mixed with modified Tyrodes-HEPES buffer in the presence and absence of various concentrations of 1,8-cineole to a final volume of 950 μL and incubated for 5 min. Then, 50 μL thrombin (1 U/mL) was added to initiate clot formation. A blunt glass capillary was placed inside the tube around which the clot was formed, and the clot retraction was monitored over a period of 2 h at room temperature. After 2 h, the remaining clot weight was measured as a marker for clot retraction.

### 4.8. In Vitro Thrombus Formation

Human whole blood was incubated with 5 μM of a lipophilic dye, DiOC6 (3,3′-Dihexyloxacarbocyanine Iodide) (Sigma Aldrich, Gillingham, UK) at 30°C for 30 min. Vena8 BioChip (Cellix Ltd., Ireland) microfluidic channels were coated with collagen (400 µg/mL) for one hour. Following blocking with 1% (*w/v*) bovine serum albumin for one hour, the human whole blood pre-incubated with a vehicle control or various concentrations (6.25, 12.5 and 50μM) of 1,8-cineole for 5 min was perfused through the collagen-coated microfluidic channels at a shear stress of 20 dynes/cm^2^ for 10 min. The level of thrombus formation was observed using a Nikon A1-R confocal microscope using 20× objective. Fluorescence images of thrombi were captured every 30 s continuously for 10 min. The median fluorescence intensity of thrombi was calculated using NIS Elements software (Nikon, Tokyo, Japan) and the images were analysed using ImageJ software (National Institute of Health, Bethesda, MD, USA).

### 4.9. Tail Bleeding Assay

This experiment has been approved by the University of Reading Research Ethics Committee and the British Home Office (PPL 7709063). Briefly, 12 weeks old C57BL/6 mice (Envigo, London, UK) were anaesthetised [using ketamine (80 mg/kg) and xylazine (5 mg/kg)] via intraperitoneal route and the mice were placed on a heated pad (37°C). After 20 min, a vehicle control [0.01% (*v/v*) ethanol] or 1,8-cineole (12.5 µM and 6.25 µM—final concentration—calculated based on the estimated volume of blood using mouse weight) was administered via femoral artery and incubated for 5 min. Then, the distal 3 mm segment of the tail tip was dissected using a scalpel blade and the tail tip was placed in sterile saline at 37 °C and the time taken to cessation of bleeding was measured up to 20 min at which point the assay was terminated. 

### 4.10. LDH Cytotoxicity Assay

LDH cytotoxicity assay was performed using Pierce LDH Cytotoxicity Assay Kit (Thermo Fisher, Gloucester, UK) according to our optimized protocols for platelets. Human isolated platelets were incubated with various concentrations of 1,8-cineole or a positive control (a detergent provided in the kit) for 5 min. To this, the reaction mixture (provided in the kit) was added and incubated for 30 min at 37°C. Following incubation, a stop solution that was also provided in the kit was added to terminate the reaction and the absorbance of this mixture was read at 490 nm and 650 nm using spectrophotometer (Molecular devices, Wokingham, UK). 

### 4.11. Immunoblotting Analysis

SDS-PAGE and immunoblotting analyses were performed using standard protocols [56,57,58]. Human isolated platelets were treated with different concentrations of 1,8-cineole and a vehicle control [0.01% (*v/v*) ethanol] for 5 min and an agonist was added to trigger platelet activation. After 5 min, the activation was stopped by adding reducing sample treatment buffer and the obtained platelet lysates were used for SDS-PAGE followed by immunoblotting experiments with various antibodies. The mouse anti-human 14-3-3ζ antibody (Santa Cruz Biotechnology, Santa Cruz, CA, USA) was used to detect the protein, 14-3-3ζ as a loading control in immunoblots. The Cy5-conjugated goat anti-rabbit and anti-mouse IgG were used as secondary antibodies in these experiments.

### 4.12. Quantification of cAMP Levels in Platelets

The cAMP levels in platelets were quantified using cAMP ELISA quantification kit (Enzo Life sciences, Exeter, UK) according to the manufacturer’s instructions. Human isolated platelets were treated with a vehicle control or different concentrations of 1,8-cineole prior to measuring the level of cAMP using a cAMP ELISA kit. The amount of cAMP was quantified using the standard curve which was plotted using the control samples provided in the kit.

### 4.13. Statistical Analysis

All the data are represented as mean ± SEM. The statistical significance was determined using one-way ANOVA except for the tail bleeding assay where the data were analysed using a non-parametric Kruskal–Wallis test. All the statistical analyses were performed using GraphPad Prism 7 software (GraphPad Software Inc., San Diego, CA, USA).

## 5. Conclusions

In conclusion, essential oils extracted from medicinal plants have been extensively used for the treatment of various diseases and they are becoming as alternative therapeutics worldwide [5,6]. 1,8-cineole has been used for many years for it is anti-inflammatory and antioxidant effects [12]. Several studies have shown that 1,8-cineole is effective and safe in the treatment of several diseases such bronchitis and inflammatory conditions [12]. Indeed, the clinical grade 1,8-cineole has been approved as a drug to treat some of these conditions. The results obtained in this study demonstrate that 1,8-cineole has potent inhibitory effects on human platelet function in isolated platelets, PRP (in the presence of plasma proteins) and in whole blood. As low as 6.25 μM may be sufficient to reduce unwarranted platelet activation in the circulation. Therefore, 1,8-cineole could be beneficial in preventing and treating thrombotic diseases. Further studies to investigate the therapeutic potential and safety profile of 1,8-cineole in humans may aid in the design and development of novel anti-thrombotic strategies using this compound as a source or template. Due to its numerous beneficial effects and pharmacological properties, this may be used as a safe and effective anti-thrombotic agent to control thrombotic diseases.
